# Supplementary material for: The Real-Time Monitoring of the Laser-Induced Functionalization of Transparent Conductive Oxide Films
Source: Nanomaterials (Basel). 2023 Oct 5;13(19):2706. doi: 10.3390/nano13192706 (PMC10574555; doi:10.3390/nano13192706)
Supplement: Supplementary file 1 [file nanomaterials-13-02706-s001.zip › SuppMater.pdf]

The Real-Time Monitoring of the Laser-induced Functionalization of Transparent Conductive Oxide Films

Takuya Hosokai [1,*], Junichi Nomoto [2]

Supplementary Material Section

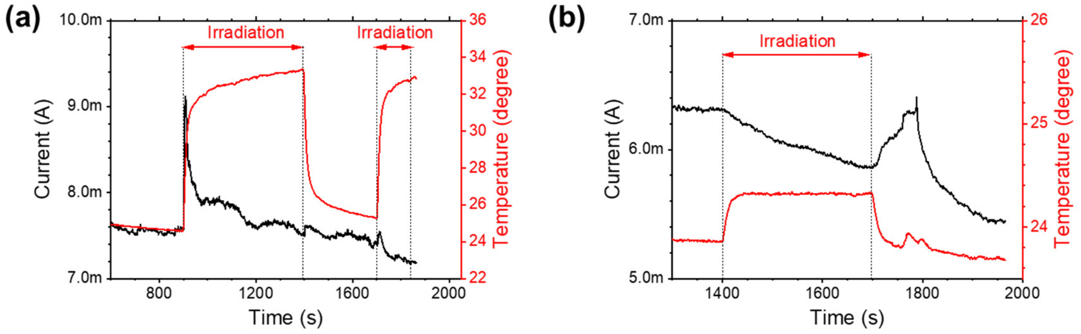

Figure S1. Temporal results of the temperature and current of the ICO:H/PET films under FHG irradiation. (a) laser repetition rate was 1 kH (a) and 100 Hz (b).
